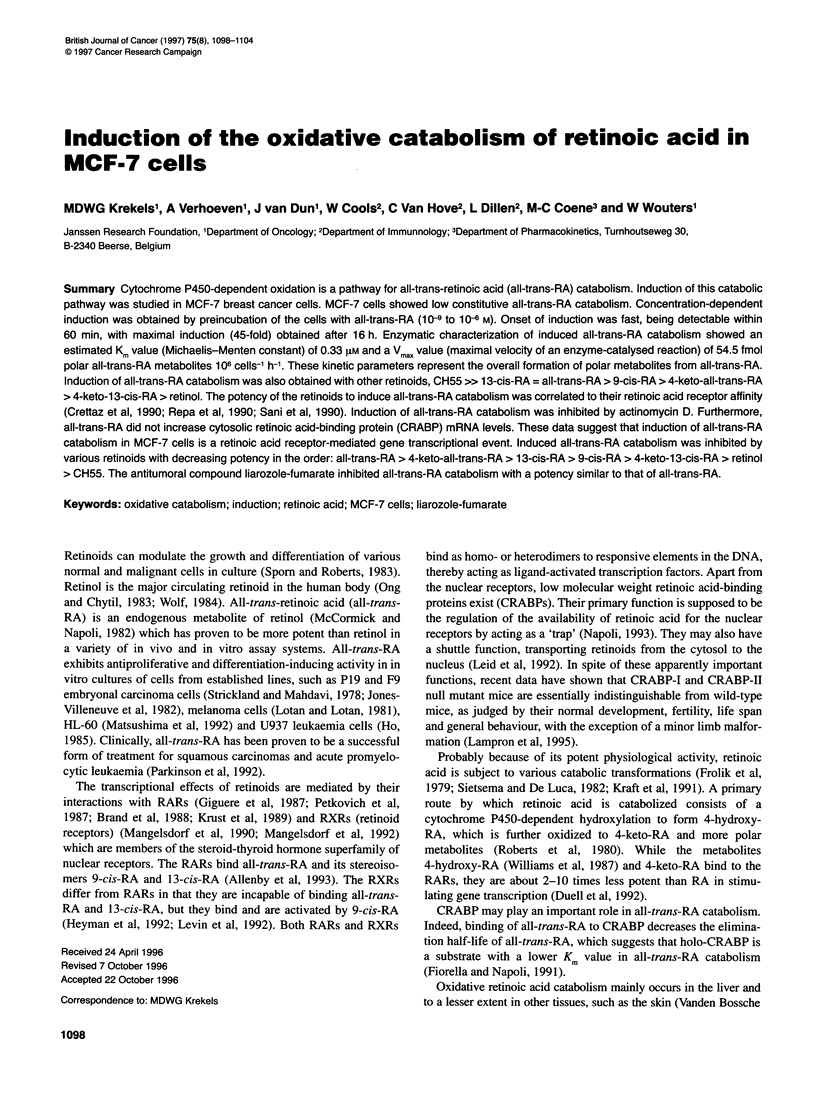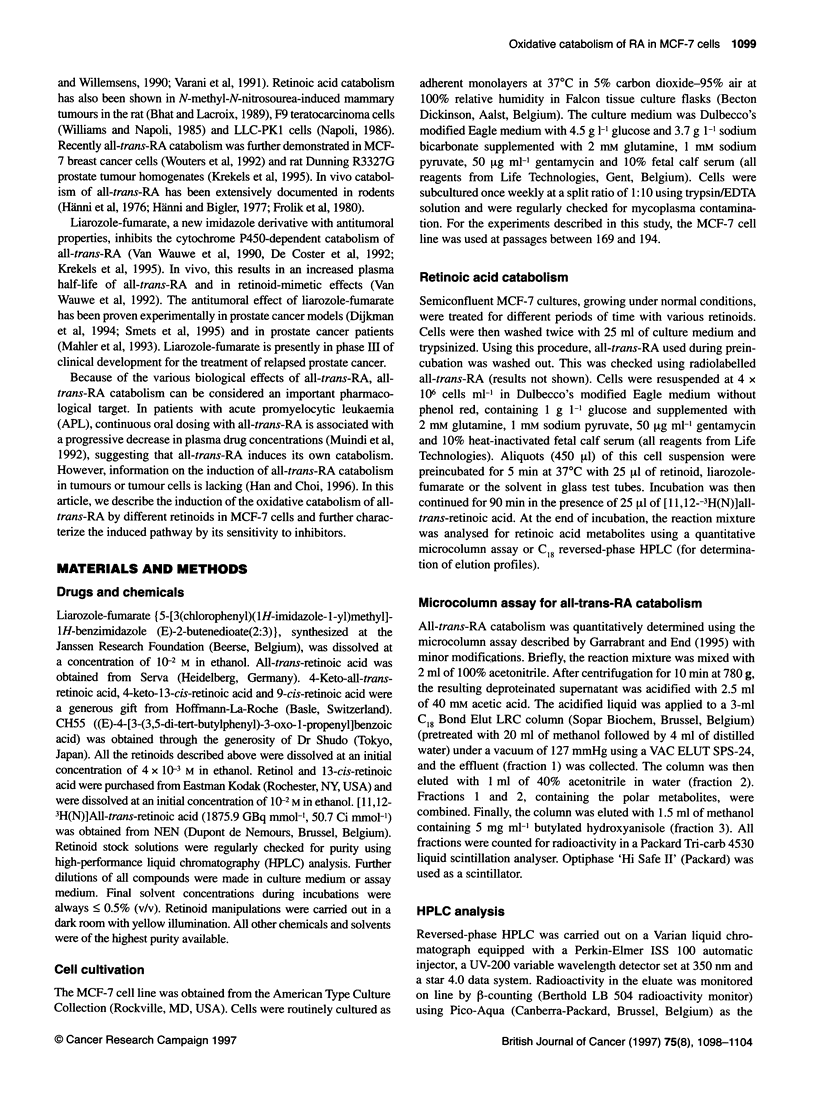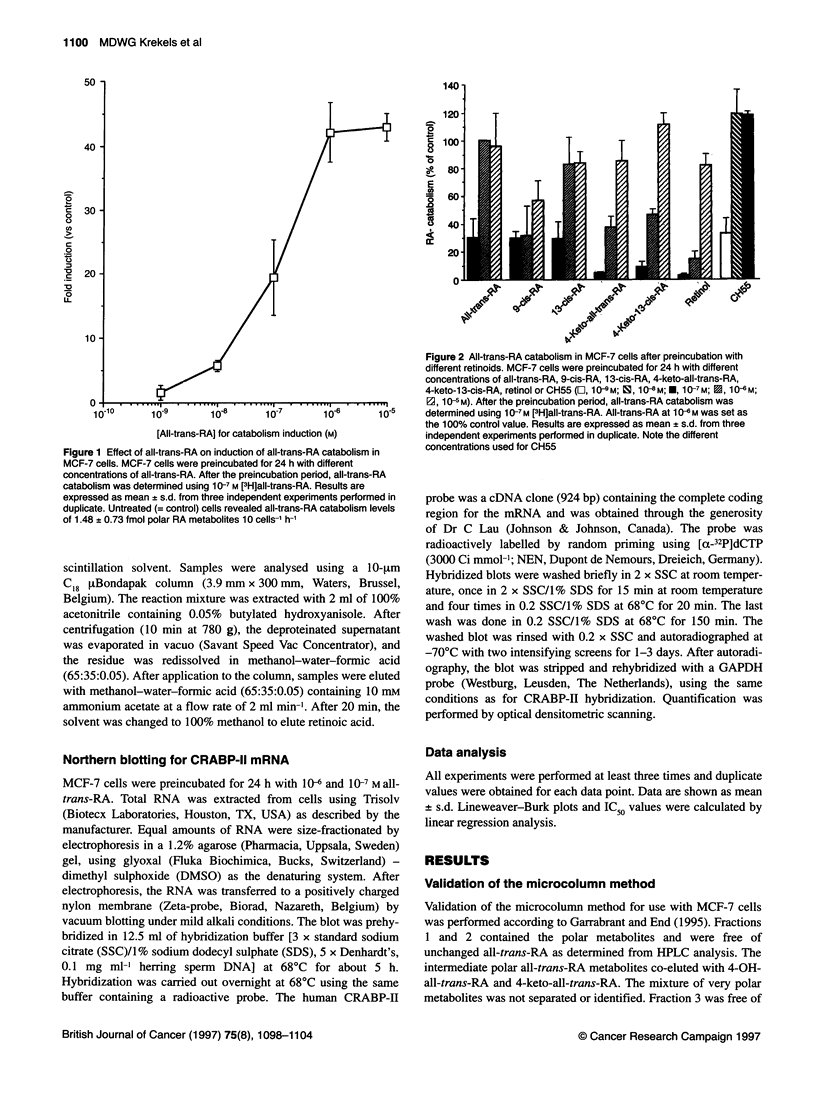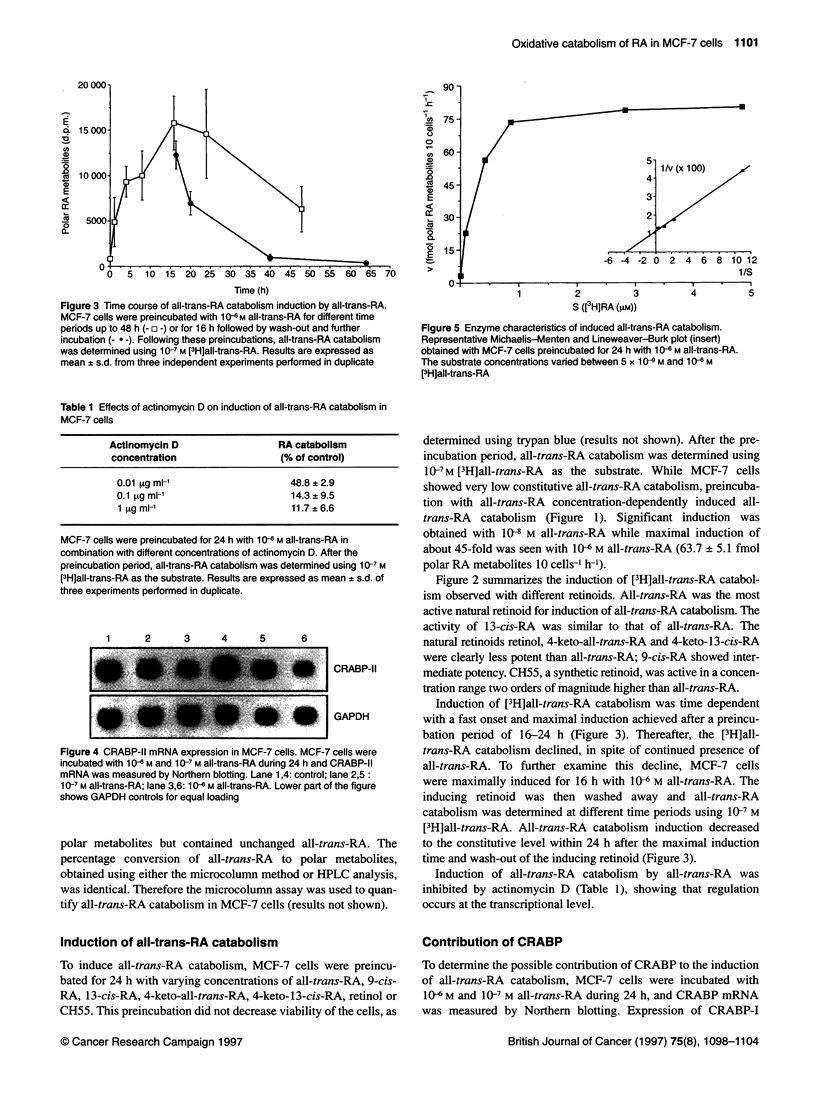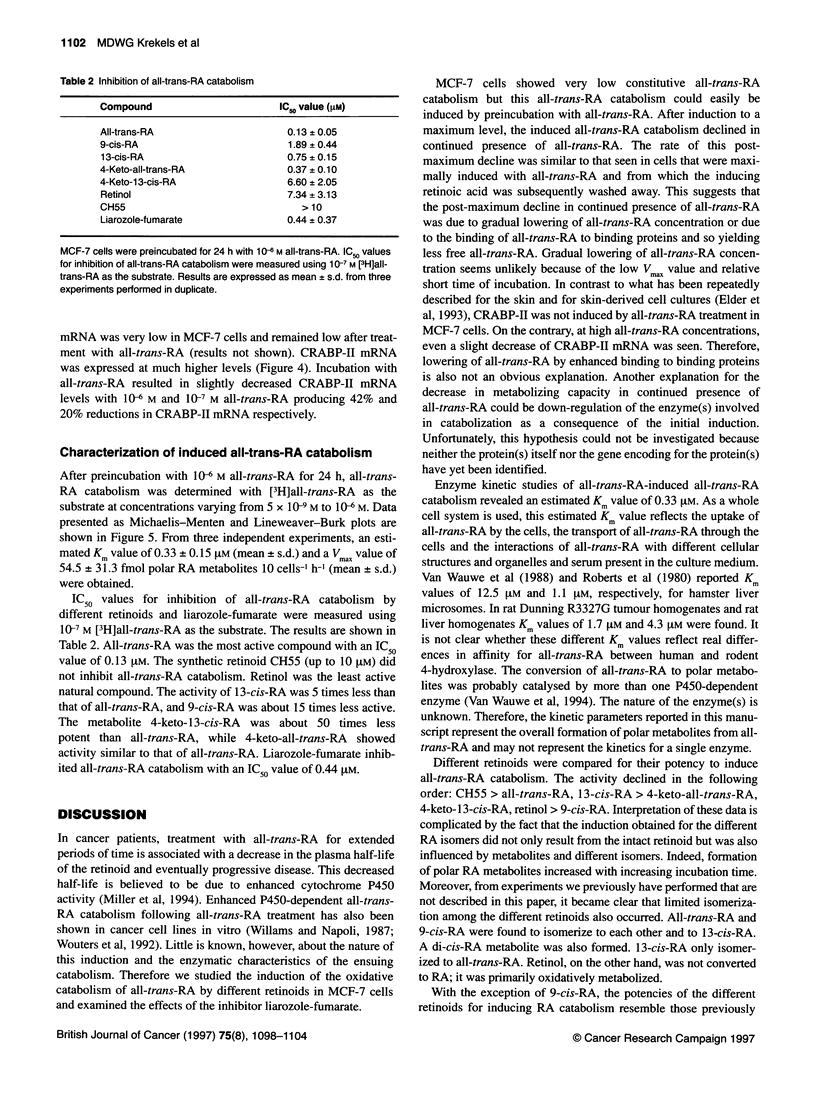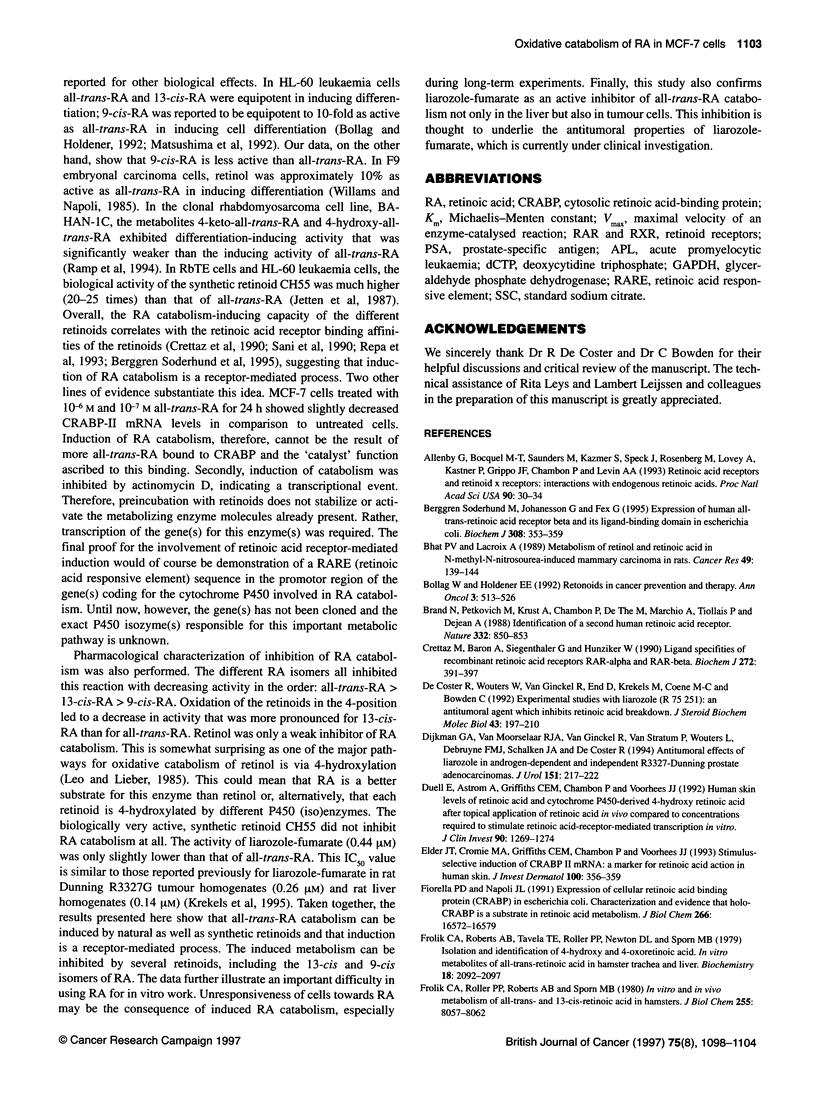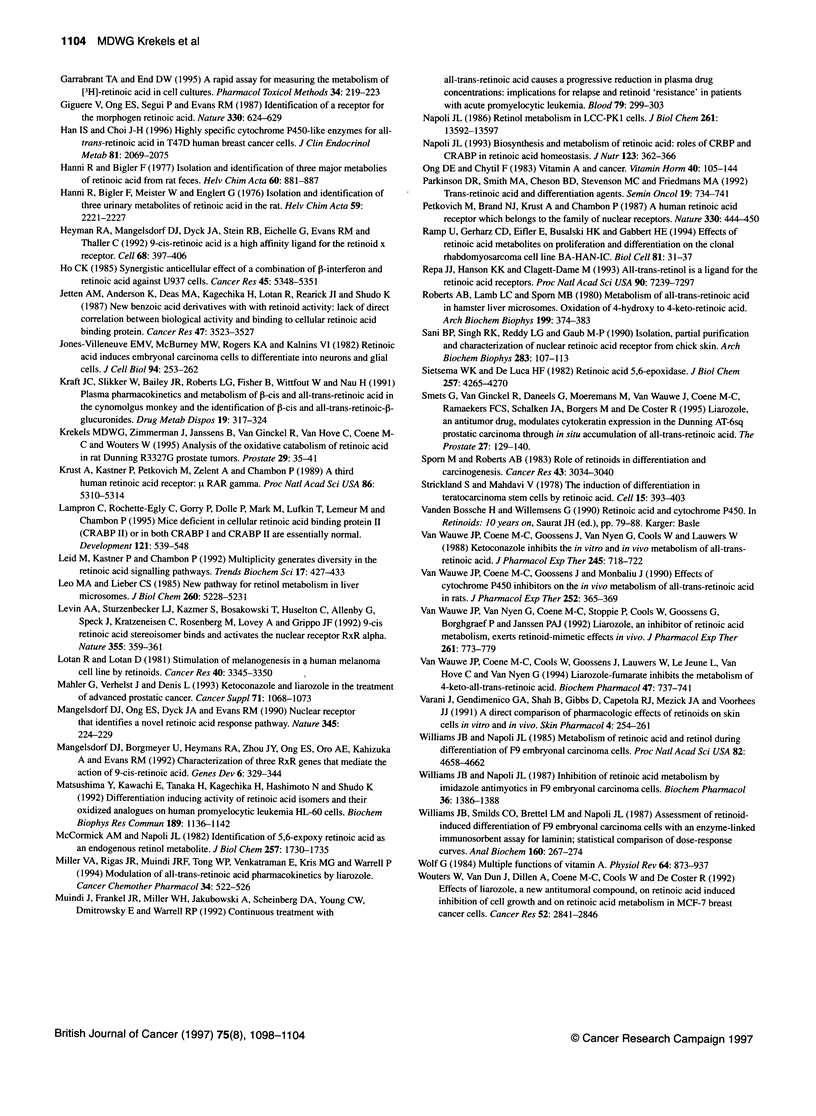# Induction of the oxidative catabolism of retinoid acid in MCF-7 cells.

**DOI:** 10.1038/bjc.1997.190

**Published:** 1997

**Authors:** M. D. Krekels, A. Verhoeven, J. van Dun, W. Cools, C. Van Hove, L. Dillen, M. C. Coene, W. Wouters

**Affiliations:** Janssen Research Foundation, Department of Oncology, Beerse, Belgium.

## Abstract

**Images:**


British Journal of Cancer (1997) 75(8), 1098–1104
© 1997 Cancer Research Campaign

### Induction of the oxidative catabolism of retinoic acid in MCF-7 cells

**MDWG Krekels[1], A Verhoeven[1], J van Dun[1], W Cools[2], C Van Hove[2], L Dillen[2], M-C Coene[3] and W Wouters[1]**

Janssen Research Foundation, [1]Department of Oncology; [2]Department of Immunnology; [3]Department of Pharmacokinetics, Turnhoutseweg 30, B-2340 Beerse, Belgium

**Summary** Cytochrome P450-dependent oxidation is a pathway for all-trans-retinoic acid (all-trans-RA) catabolism. Induction of this catabolic pathway was studied in MCF-7 breast cancer cells. MCF-7 cells showed low constitutive all-trans-RA catabolism. Concentration-dependent induction was obtained by preincubation of the cells with all-trans-RA ($10^{-9}$ to $10^{-6}$ M). Onset of induction was fast, being detectable within 60 min, with maximal induction (45-fold) obtained after 16 h. Enzymatic characterization of induced all-trans-RA catabolism showed an estimated $K_m$ value (Michaelis–Menten constant) of 0.33 μM and a $V_{max}$ value (maximal velocity of an enzyme-catalysed reaction) of 54.5 fmol polar all-trans-RA metabolites $10^6$ cells$^{-1}$ h$^{-1}$. These kinetic parameters represent the overall formation of polar metabolites from all-trans-RA. Induction of all-trans-RA catabolism was also obtained with other retinoids, CH55 >> 13-cis-RA = all-trans-RA > 9-cis-RA > 4-keto-all-trans-RA > 4-keto-13-cis-RA > retinol. The potency of the retinoids to induce all-trans-RA catabolism was correlated to their retinoic acid receptor affinity (Crettaz et al, 1990; Repa et al, 1990; Sani et al, 1990). Induction of all-trans-RA catabolism was inhibited by actinomycin D. Furthermore, all-trans-RA did not increase cytosolic retinoic acid-binding protein (CRABP) mRNA levels. These data suggest that induction of all-trans-RA catabolism in MCF-7 cells is a retinoic acid receptor-mediated gene transcriptional event. Induced all-trans-RA catabolism was inhibited by various retinoids with decreasing potency in the order: all-trans-RA > 4-keto-all-trans-RA > 13-cis-RA > 9-cis-RA > 4-keto-13-cis-RA > retinol > CH55. The antitumoral compound liarozole-fumarate inhibited all-trans-RA catabolism with a potency similar to that of all-trans-RA.

**Keywords:** oxidative catabolism; induction; retinoic acid; MCF-7 cells; liarozole-fumarate

Retinoids can modulate the growth and differentiation of various normal and malignant cells in culture (Sporn and Roberts, 1983). Retinol is the major circulating retinoid in the human body (Ong and Chytil, 1983; Wolf, 1984). All-*trans*-retinoic acid (all-*trans*-RA) is an endogenous metabolite of retinol (McCormick and Napoli, 1982) which has proven to be more potent than retinol in a variety of in vivo and in vitro assay systems. All-*trans*-RA exhibits antiproliferative and differentiation-inducing activity in in vitro cultures of cells from established lines, such as P19 and F9 embryonal carcinoma cells (Strickland and Mahdavi, 1978; Jones-Villeneuve et al, 1982), melanoma cells (Lotan and Lotan, 1981), HL-60 (Matsushima et al, 1992) and U937 leukaemia cells (Ho, 1985). Clinically, all-*trans*-RA has been proven to be a successful form of treatment for squamous carcinomas and acute promyelocytic leukaemia (Parkinson et al, 1992).

The transcriptional effects of retinoids are mediated by their interactions with RARs (Giguere et al, 1987; Petkovich et al, 1987; Brand et al, 1988; Krust et al, 1989) and RXRs (retinoid receptors) (Mangelsdorf et al, 1990; Mangelsdorf et al, 1992) which are members of the steroid-thyroid hormone superfamily of nuclear receptors. The RARs bind all-*trans*-RA and its stereoisomers 9-*cis*-RA and 13-*cis*-RA (Allenby et al, 1993). The RXRs differ from RARs in that they are incapable of binding all-*trans*-RA and 13-*cis*-RA, but they bind and are activated by 9-*cis*-RA (Heyman et al, 1992; Levin et al, 1992). Both RARs and RXRs bind as homo- or heterodimers to responsive elements in the DNA, thereby acting as ligand-activated transcription factors. Apart from the nuclear receptors, low molecular weight retinoic acid-binding proteins exist (CRABPs). Their primary function is supposed to be the regulation of the availability of retinoic acid for the nuclear receptors by acting as a 'trap' (Napoli, 1993). They may also have a shuttle function, transporting retinoids from the cytosol to the nucleus (Leid et al, 1992). In spite of these apparently important functions, recent data have shown that CRABP-I and CRABP-II null mutant mice are essentially indistinguishable from wild-type mice, as judged by their normal development, fertility, life span and general behaviour, with the exception of a minor limb malformation (Lampron et al, 1995).

Received 24 April 1996
Revised 7 October 1996
Accepted 22 October 1996

Correspondence to: MDWG Krekels

Probably because of its potent physiological activity, retinoic acid is subject to various catabolic transformations (Frolik et al, 1979; Sietsema and De Luca, 1982; Kraft et al, 1991). A primary route by which retinoic acid is catabolized consists of a cytochrome P450-dependent hydroxylation to form 4-hydroxy-RA, which is further oxidized to 4-keto-RA and more polar metabolites (Roberts et al, 1980). While the metabolites 4-hydroxy-RA (Williams et al, 1987) and 4-keto-RA bind to the RARs, they are about 2–10 times less potent than RA in stimulating gene transcription (Duell et al, 1992).

CRABP may play an important role in all-*trans*-RA catabolism. Indeed, binding of all-*trans*-RA to CRABP decreases the elimination half-life of all-*trans*-RA, which suggests that holo-CRABP is a substrate with a lower $K_m$ value in all-*trans*-RA catabolism (Fiorella and Napoli, 1991).

Oxidative retinoic acid catabolism mainly occurs in the liver and to a lesser extent in other tissues, such as the skin (Vanden Bossche and Willemsens, 1990; Varani et al, 1991). Retinoic acid catabolism has also been shown in *N*-methyl-*N*-nitrosourea-induced mammary tumours in the rat (Bhat and Lacroix, 1989), F9 teratocarcinoma cells (Williams and Napoli, 1985) and LLC-PK1 cells (Napoli, 1986). Recently all-*trans*-RA catabolism was further demonstrated in MCF-7 breast cancer cells (Wouters et al, 1992) and rat Dunning R3327G prostate tumour homogenates (Krekels et al, 1995). In vivo catabolism of all-*trans*-RA has been extensively documented in rodents (Hänni et al, 1976; Hänni and Bigler, 1977; Frolik et al, 1980).

Liarozole-fumarate, a new imidazole derivative with antitumoral properties, inhibits the cytochrome P450-dependent catabolism of all-*trans*-RA (Van Wauwe et al, 1990, De Coster et al, 1992; Krekels et al, 1995). In vivo, this results in an increased plasma half-life of all-*trans*-RA and in retinoid-mimetic effects (Van Wauwe et al, 1992). The antitumoral effect of liarozole-fumarate has been proven experimentally in prostate cancer models (Dijkman et al, 1994; Smets et al, 1995) and in prostate cancer patients (Mahler et al, 1993). Liarozole-fumarate is presently in phase III of clinical development for the treatment of relapsed prostate cancer.

Because of the various biological effects of all-*trans*-RA, all-*trans*-RA catabolism can be considered an important pharmacological target. In patients with acute promyelocytic leukaemia (APL), continuous oral dosing with all-*trans*-RA is associated with a progressive decrease in plasma drug concentrations (Muindi et al, 1992), suggesting that all-*trans*-RA induces its own catabolism. However, information on the induction of all-*trans*-RA catabolism in tumours or tumour cells is lacking (Han and Choi, 1996). In this article, we describe the induction of the oxidative catabolism of all-*trans*-RA by different retinoids in MCF-7 cells and further characterize the induced pathway by its sensitivity to inhibitors.

### MATERIALS AND METHODS

### Drugs and chemicals

Liarozole-fumarate {5-[3(chlorophenyl)(1*H*-imidazole-1-yl)methyl]-1*H*-benzimidazole (E)-2-butenedioate(2:3)}, synthesized at the Janssen Research Foundation (Beerse, Belgium), was dissolved at a concentration of $10^{-2}$ M in ethanol. All-*trans*-retinoic acid was obtained from Serva (Heidelberg, Germany). 4-Keto-all-*trans*-retinoic acid, 4-keto-13-*cis*-retinoic acid and 9-*cis*-retinoic acid were a generous gift from Hoffmann-La-Roche (Basle, Switzerland). CH55 ((E)-4-[3-(3,5-di-tert-butylphenyl)-3-oxo-1-propenyl]benzoic acid) was obtained through the generosity of Dr Shudo (Tokyo, Japan). All the retinoids described above were dissolved at an initial concentration of $4 \times 10^{-3}$ M in ethanol. Retinol and 13-*cis*-retinoic acid were purchased from Eastman Kodak (Rochester, NY, USA) and were dissolved at an initial concentration of $10^{-2}$ M in ethanol. [11,12-$^{3}$H(N)]All-*trans*-retinoic acid (1875.9 GBq mmol$^{-1}$, 50.7 Ci mmol$^{-1}$) was obtained from NEN (Dupont de Nemours, Brussel, Belgium). Retinoid stock solutions were regularly checked for purity using high-performance liquid chromatography (HPLC) analysis. Further dilutions of all compounds were made in culture medium or assay medium. Final solvent concentrations during incubations were always $\leq$ 0.5% (v/v). Retinoid manipulations were carried out in a dark room with yellow illumination. All other chemicals and solvents were of the highest purity available.

### Cell cultivation

The MCF-7 cell line was obtained from the American Type Culture Collection (Rockville, MD, USA). Cells were routinely cultured as adherent monolayers at 37°C in 5% carbon dioxide–95% air at 100% relative humidity in Falcon tissue culture flasks (Becton Dickinson, Aalst, Belgium). The culture medium was Dulbecco's modified Eagle medium with 4.5 g l$^{-1}$ glucose and 3.7 g l$^{-1}$ sodium bicarbonate supplemented with 2 mM glutamine, 1 mM sodium pyruvate, 50 μg ml$^{-1}$ gentamycin and 10% fetal calf serum (all reagents from Life Technologies, Gent, Belgium). Cells were subcultured once weekly at a split ratio of 1:10 using trypsin/EDTA solution and were regularly checked for mycoplasma contamination. For the experiments described in this study, the MCF-7 cell line was used at passages between 169 and 194.

### Retinoic acid catabolism

Semiconfluent MCF-7 cultures, growing under normal conditions, were treated for different periods of time with various retinoids. Cells were then washed twice with 25 ml of culture medium and trypsinized. Using this procedure, all-*trans*-RA used during preincubation was washed out. This was checked using radiolabelled all-*trans*-RA (results not shown). Cells were resuspended at 4 × $10^{6}$ cells ml$^{-1}$ in Dulbecco's modified Eagle medium without phenol red, containing 1 g l$^{-1}$ glucose and supplemented with 2 mM glutamine, 1 mM sodium pyruvate, 50 μg ml$^{-1}$ gentamycin and 10% heat-inactivated fetal calf serum (all reagents from Life Technologies). Aliquots (450 μl) of this cell suspension were preincubated for 5 min at 37°C with 25 μl of retinoid, liarozole-fumarate or the solvent in glass test tubes. Incubation was then continued for 90 min in the presence of 25 μl of [11,12-$^{3}$H(N)]all-*trans*-retinoic acid. At the end of incubation, the reaction mixture was analysed for retinoic acid metabolites using a quantitative microcolumn assay or $C_{18}$ reversed-phase HPLC (for determination of elution profiles).

### Microcolumn assay for all-trans-RA catabolism

All-*trans*-RA catabolism was quantitatively determined using the microcolumn assay described by Garrabrant and End (1995) with minor modifications. Briefly, the reaction mixture was mixed with 2 ml of 100% acetonitrile. After centrifugation for 10 min at 780 **g**, the resulting deproteinated supernatant was acidified with 2.5 ml of 40 mM acetic acid. The acidified liquid was applied to a 3-ml $C_{18}$ Bond Elut LRC column (Sopar Biochem, Brussel, Belgium) (pretreated with 20 ml of methanol followed by 4 ml of distilled water) under a vacuum of 127 mmHg using a VAC ELUT SPS-24, and the effluent (fraction 1) was collected. The column was then eluted with 1 ml of 40% acetonitrile in water (fraction 2). Fractions 1 and 2, containing the polar metabolites, were combined. Finally, the column was eluted with 1.5 ml of methanol containing 5 mg ml$^{-1}$ butylated hydroxyanisole (fraction 3). All fractions were counted for radioactivity in a Packard Tri-carb 4530 liquid scintillation analyser. Optiphase 'Hi Safe II' (Packard) was used as a scintillator.

### HPLC analysis

Reversed-phase HPLC was carried out on a Varian liquid chromatograph equipped with a Perkin-Elmer ISS 100 automatic injector, a UV-200 variable wavelength detector set at 350 nm and a star 4.0 data system. Radioactivity in the eluate was monitored on line by β-counting (Berthold LB 504 radioactivity monitor) using Pico-Aqua (Canberra-Packard, Brussel, Belgium) as the

© Cancer Research Campaign 1997

**Figure 1** Effect of all-trans-RA on induction of all-trans-RA catabolism in MCF-7 cells. MCF-7 cells were preincubated for 24 h with different concentrations of all-trans-RA. After the preincubation period, all-trans-RA catabolism was determined using $10^{-7}$ M [$^3$H]all-trans-RA. Results are expressed as mean ± s.d. from three independent experiments performed in duplicate. Untreated (= control) cells revealed all-trans-RA catabolism levels of 1.48 ± 0.73 fmol polar RA metabolites 10 cells$^{-1}$ h$^{-1}$

**Figure 2** All-trans-RA catabolism in MCF-7 cells after preincubation with different retinoids. MCF-7 cells were preincubated for 24 h with different concentrations of all-trans-RA, 9-cis-RA, 13-cis-RA, 4-keto-all-trans-RA, 4-keto-13-cis-RA, retinol or CH55 (□, $10^{-9}$ M; ▧, $10^{-8}$ M; ■, $10^{-7}$ M; ▨, $10^{-6}$ M; ▨, $10^{-5}$ M). After the preincubation period, all-trans-RA catabolism was determined using $10^{-7}$ M [$^3$H]all-trans-RA. All-trans-RA at $10^{-6}$ M was set as the 100% control value. Results are expressed as mean ± s.d. from three independent experiments performed in duplicate. Note the different concentrations used for CH55

scintillation solvent. Samples were analysed using a 10-µm $C_{18}$ µBondapak column (3.9 mm × 300 mm, Waters, Brussel, Belgium). The reaction mixture was extracted with 2 ml of 100% acetonitrile containing 0.05% butylated hydroxyanisole. After centrifugation (10 min at 780 g), the deproteinated supernatant was evaporated in vacuo (Savant Speed Vac Concentrator), and the residue was redissolved in methanol–water–formic acid (65:35:0.05). After application to the column, samples were eluted with methanol–water–formic acid (65:35:0.05) containing 10 mM ammonium acetate at a flow rate of 2 ml min$^{-1}$. After 20 min, the solvent was changed to 100% methanol to elute retinoic acid.

### Northern blotting for CRABP-II mRNA

MCF-7 cells were preincubated for 24 h with $10^{-6}$ and $10^{-7}$ M all-*trans*-RA. Total RNA was extracted from cells using Trisolv (Biotecx Laboratories, Houston, TX, USA) as described by the manufacturer. Equal amounts of RNA were size-fractionated by electrophoresis in a 1.2% agarose (Pharmacia, Uppsala, Sweden) gel, using glyoxal (Fluka Biochimica, Bucks, Switzerland) – dimethyl sulphoxide (DMSO) as the denaturing system. After electrophoresis, the RNA was transferred to a positively charged nylon membrane (Zeta-probe, Biorad, Nazareth, Belgium) by vacuum blotting under mild alkali conditions. The blot was prehybridized in 12.5 ml of hybridization buffer [3 × standard sodium citrate (SSC)/1% sodium dodecyl sulphate (SDS), 5 × Denhardt's, 0.1 mg ml$^{-1}$ herring sperm DNA] at 68°C for about 5 h. Hybridization was carried out overnight at 68°C using the same buffer containing a radioactive probe. The human CRABP-II probe was a cDNA clone (924 bp) containing the complete coding region for the mRNA and was obtained through the generosity of Dr C Lau (Johnson & Johnson, Canada). The probe was radioactively labelled by random priming using [α-$^{32}$P]dCTP (3000 Ci mmol$^{-1}$; NEN, Dupont de Nemours, Dreieich, Germany). Hybridized blots were washed briefly in 2 × SSC at room temperature, once in 2 × SSC/1% SDS for 15 min at room temperature and four times in 0.2 SSC/1% SDS at 68°C for 20 min. The last wash was done in 0.2 SSC/1% SDS at 68°C for 150 min. The washed blot was rinsed with 0.2 × SSC and autoradiographed at –70°C with two intensifying screens for 1–3 days. After autoradiography, the blot was stripped and rehybridized with a GAPDH probe (Westburg, Leusden, The Netherlands), using the same conditions as for CRABP-II hybridization. Quantification was performed by optical densitometric scanning.

### Data analysis

All experiments were performed at least three times and duplicate values were obtained for each data point. Data are shown as mean ± s.d. Lineweaver–Burk plots and $IC_{50}$ values were calculated by linear regression analysis.

### RESULTS

### Validation of the microcolumn method

Validation of the microcolumn method for use with MCF-7 cells was performed according to Garrabrant and End (1995). Fractions 1 and 2 contained the polar metabolites and were free of unchanged all-*trans*-RA as determined from HPLC analysis. The intermediate polar all-*trans*-RA metabolites co-eluted with 4-OH-all-*trans*-RA and 4-keto-all-*trans*-RA. The mixture of very polar metabolites was not separated or identified. Fraction 3 was free of

© Cancer Research Campaign 1997

**Figure 3** Time course of all-trans-RA catabolism induction by all-trans-RA. MCF-7 cells were preincubated with $10^{-6}$ M all-trans-RA for different time periods up to 48 h (- □ -) or for 16 h followed by wash-out and further incubation (- • -). Following these preincubations, all-trans-RA catabolism was determined using $10^{-7}$ M [$^3$H]all-trans-RA. Results are expressed as mean ± s.d. from three independent experiments performed in duplicate

**Figure 5** Enzyme characteristics of induced all-trans-RA catabolism. Representative Michaelis–Menten and Lineweaver–Burk plot (insert) obtained with MCF-7 cells preincubated for 24 h with $10^{-6}$ M all-trans-RA. The substrate concentrations varied between 5 × $10^{-9}$ M and $10^{-6}$ M [$^3$H]all-trans-RA

**Table 1** Effects of actinomycin D on induction of all-trans-RA catabolism in MCF-7 cells

| Actinomycin D concentration | RA catabolism (% of control) |
|---|---|
| 0.01 μg ml$^{-1}$ | 48.8 ± 2.9 |
| 0.1 μg ml$^{-1}$ | 14.3 ± 9.5 |
| 1 μg ml$^{-1}$ | 11.7 ± 6.6 |

MCF-7 cells were preincubated for 24 h with $10^{-6}$ M all-trans-RA in combination with different concentrations of actinomycin D. After the preincubation period, all-trans-RA catabolism was determined using $10^{-7}$ M [$^3$H]all-trans-RA as the substrate. Results are expressed as mean ± s.d. of three experiments performed in duplicate.

**Figure 4** CRABP-II mRNA expression in MCF-7 cells. MCF-7 cells were incubated with $10^{-6}$ M and $10^{-7}$ M all-trans-RA during 24 h and CRABP-II mRNA was measured by Northern blotting. Lane 1,4: control; lane 2,5 : $10^{-7}$ M all-trans-RA; lane 3,6: $10^{-6}$ M all-trans-RA. Lower part of the figure shows GAPDH controls for equal loading polar metabolites but contained unchanged all-*trans*-RA. The percentage conversion of all-*trans*-RA to polar metabolites, obtained using either the microcolumn method or HPLC analysis, was identical. Therefore the microcolumn assay was used to quantify all-*trans*-RA catabolism in MCF-7 cells (results not shown).

### Induction of all-trans-RA catabolism

To induce all-*trans*-RA catabolism, MCF-7 cells were preincubated for 24 h with varying concentrations of all-*trans*-RA, 9-*cis*-RA, 13-*cis*-RA, 4-keto-all-*trans*-RA, 4-keto-13-*cis*-RA, retinol or CH55. This preincubation did not decrease viability of the cells, as determined using trypan blue (results not shown). After the preincubation period, all-*trans*-RA catabolism was determined using $10^{-7}$ M [$^3$H]all-*trans*-RA as the substrate. While MCF-7 cells showed very low constitutive all-*trans*-RA catabolism, preincubation with all-*trans*-RA concentration-dependently induced all-*trans*-RA catabolism (Figure 1). Significant induction was obtained with $10^{-8}$ M all-*trans*-RA while maximal induction of about 45-fold was seen with $10^{-6}$ M all-*trans*-RA (63.7 ± 5.1 fmol polar RA metabolites 10 cells$^{-1}$ h$^{-1}$).

Figure 2 summarizes the induction of [$^3$H]all-*trans*-RA catabolism observed with different retinoids. All-*trans*-RA was the most active natural retinoid for induction of all-*trans*-RA catabolism. The activity of 13-*cis*-RA was similar to that of all-*trans*-RA. The natural retinoids retinol, 4-keto-all-*trans*-RA and 4-keto-13-*cis*-RA were clearly less potent than all-*trans*-RA; 9-*cis*-RA showed intermediate potency. CH55, a synthetic retinoid, was active in a concentration range two orders of magnitude higher than all-*trans*-RA.

Induction of [$^3$H]all-*trans*-RA catabolism was time dependent with a fast onset and maximal induction achieved after a preincubation period of 16–24 h (Figure 3). Thereafter, the [$^3$H]all-*trans*-RA catabolism declined, in spite of continued presence of all-*trans*-RA. To further examine this decline, MCF-7 cells were maximally induced for 16 h with $10^{-6}$ M all-*trans*-RA. The inducing retinoid was then washed away and all-*trans*-RA catabolism was determined at different time periods using $10^{-7}$ M [$^3$H]all-*trans*-RA. All-*trans*-RA catabolism induction decreased to the constitutive level within 24 h after the maximal induction time and wash-out of the inducing retinoid (Figure 3).

Induction of all-*trans*-RA catabolism by all-*trans*-RA was inhibited by actinomycin D (Table 1), showing that regulation occurs at the transcriptional level.

### Contribution of CRABP

To determine the possible contribution of CRABP to the induction of all-*trans*-RA catabolism, MCF-7 cells were incubated with $10^{-6}$ M and $10^{-7}$ M all-*trans*-RA during 24 h, and CRABP mRNA was measured by Northern blotting. Expression of CRABP-I

© Cancer Research Campaign 1997

**Table 2** Inhibition of all-trans-RA catabolism

| Compound | $IC_{50}$ value (μM) |
|---|---|
| All-trans-RA | 0.13 ± 0.05 |
| 9-cis-RA | 1.89 ± 0.44 |
| 13-cis-RA | 0.75 ± 0.15 |
| 4-Keto-all-trans-RA | 0.37 ± 0.10 |
| 4-Keto-13-cis-RA | 6.60 ± 2.05 |
| Retinol | 7.34 ± 3.13 |
| CH55 | > 10 |
| Liarozole-fumarate | 0.44 ± 0.37 |

MCF-7 cells were preincubated for 24 h with $10^{-6}$ M all-trans-RA. $IC_{50}$ values for inhibition of all-trans-RA catabolism were measured using $10^{-7}$ M [$^3$H]all-trans-RA as the substrate. Results are expressed as mean ± s.d. from three experiments performed in duplicate.

mRNA was very low in MCF-7 cells and remained low after treatment with all-*trans*-RA (results not shown). CRABP-II mRNA was expressed at much higher levels (Figure 4). Incubation with all-*trans*-RA resulted in slightly decreased CRABP-II mRNA levels with $10^{-6}$ M and $10^{-7}$ M all-*trans*-RA producing 42% and 20% reductions in CRABP-II mRNA respectively.

### Characterization of induced all-trans-RA catabolism

After preincubation with $10^{-6}$ M all-*trans*-RA for 24 h, all-*trans*-RA catabolism was determined with [$^3$H]all-*trans*-RA as the substrate at concentrations varying from $5 \times 10^{-9}$ M to $10^{-6}$ M. Data presented as Michaelis–Menten and Lineweaver–Burk plots are shown in Figure 5. From three independent experiments, an estimated $K_m$ value of 0.33 ± 0.15 μM (mean ± s.d.) and a $V_{max}$ value of 54.5 ± 31.3 fmol polar RA metabolites 10 cells$^{-1}$ h$^{-1}$ (mean ± s.d.) were obtained.

$IC_{50}$ values for inhibition of all-*trans*-RA catabolism by different retinoids and liarozole-fumarate were measured using $10^{-7}$ M [$^3$H]all-*trans*-RA as the substrate. The results are shown in Table 2. All-*trans*-RA was the most active compound with an $IC_{50}$ value of 0.13 μM. The synthetic retinoid CH55 (up to 10 μM) did not inhibit all-*trans*-RA catabolism. Retinol was the least active natural compound. The activity of 13-*cis*-RA was 5 times less than that of all-*trans*-RA, and 9-*cis*-RA was about 15 times less active. The metabolite 4-keto-13-*cis*-RA was about 50 times less potent than all-*trans*-RA, while 4-keto-all-*trans*-RA showed activity similar to that of all-*trans*-RA. Liarozole-fumarate inhibited all-*trans*-RA catabolism with an $IC_{50}$ value of 0.44 μM.

### DISCUSSION

In cancer patients, treatment with all-*trans*-RA for extended periods of time is associated with a decrease in the plasma half-life of the retinoid and eventually progressive disease. This decreased half-life is believed to be due to enhanced cytochrome P450 activity (Miller et al, 1994). Enhanced P450-dependent all-*trans*-RA catabolism following all-*trans*-RA treatment has also been shown in cancer cell lines in vitro (Willams and Napoli, 1987; Wouters et al, 1992). Little is known, however, about the nature of this induction and the enzymatic characteristics of the ensuing catabolism. Therefore we studied the induction of the oxidative catabolism of all-*trans*-RA by different retinoids in MCF-7 cells and examined the effects of the inhibitor liarozole-fumarate.

MCF-7 cells showed very low constitutive all-*trans*-RA catabolism but this all-*trans*-RA catabolism could easily be induced by preincubation with all-*trans*-RA. After induction to a maximum level, the induced all-*trans*-RA catabolism declined in continued presence of all-*trans*-RA. The rate of this post-maximum decline was similar to that seen in cells that were maximally induced with all-*trans*-RA and from which the inducing retinoic acid was subsequently washed away. This suggests that the post-maximum decline in continued presence of all-*trans*-RA was due to gradual lowering of all-*trans*-RA concentration or due to the binding of all-*trans*-RA to binding proteins and so yielding less free all-*trans*-RA. Gradual lowering of all-*trans*-RA concentration seems unlikely because of the low $V_{max}$ value and relative short time of incubation. In contrast to what has been repeatedly described for the skin and for skin-derived cell cultures (Elder et al, 1993), CRABP-II was not induced by all-*trans*-RA treatment in MCF-7 cells. On the contrary, at high all-*trans*-RA concentrations, even a slight decrease of CRABP-II mRNA was seen. Therefore, lowering of all-*trans*-RA by enhanced binding to binding proteins is also not an obvious explanation. Another explanation for the decrease in metabolizing capacity in continued presence of all-*trans*-RA could be down-regulation of the enzyme(s) involved in catabolization as a consequence of the initial induction. Unfortunately, this hypothesis could not be investigated because neither the protein(s) itself nor the gene encoding for the protein(s) have yet been identified.

Enzyme kinetic studies of all-*trans*-RA-induced all-*trans*-RA catabolism revealed an estimated $K_m$ value of 0.33 μM. As a whole cell system is used, this estimated $K_m$ value reflects the uptake of all-*trans*-RA by the cells, the transport of all-*trans*-RA through the cells and the interactions of all-*trans*-RA with different cellular structures and organelles and serum present in the culture medium. Van Wauwe et al (1988) and Roberts et al (1980) reported $K_m$ values of 12.5 μM and 1.1 μM, respectively, for hamster liver microsomes. In rat Dunning R3327G tumour homogenates and rat liver homogenates $K_m$ values of 1.7 μM and 4.3 μM were found. It is not clear whether these different $K_m$ values reflect real differences in affinity for all-*trans*-RA between human and rodent 4-hydroxylase. The conversion of all-*trans*-RA to polar metabolites was probably catalysed by more than one P450-dependent enzyme (Van Wauwe et al, 1994). The nature of the enzyme(s) is unknown. Therefore, the kinetic parameters reported in this manuscript represent the overall formation of polar metabolites from all-*trans*-RA and may not represent the kinetics for a single enzyme.

Different retinoids were compared for their potency to induce all-*trans*-RA catabolism. The activity declined in the following order: CH55 > all-*trans*-RA, 13-*cis*-RA > 4-keto-all-*trans*-RA, 4-keto-13-*cis*-RA, retinol > 9-*cis*-RA. Interpretation of these data is complicated by the fact that the induction obtained for the different RA isomers did not only result from the intact retinoid but was also influenced by metabolites and different isomers. Indeed, formation of polar RA metabolites increased with increasing incubation time. Moreover, from experiments we previously have performed that are not described in this paper, it became clear that limited isomerization among the different retinoids also occurred. All-*trans*-RA and 9-*cis*-RA were found to isomerize to each other and to 13-*cis*-RA. A di-*cis*-RA metabolite was also formed. 13-*cis*-RA only isomerized to all-*trans*-RA. Retinol, on the other hand, was not converted to RA; it was primarily oxidatively metabolized.

With the exception of 9-*cis*-RA, the potencies of the different retinoids for inducing RA catabolism resemble those previously

© Cancer Research Campaign 1997

reported for other biological effects. In HL-60 leukaemia cells all-*trans*-RA and 13-*cis*-RA were equipotent in inducing differentiation; 9-*cis*-RA was reported to be equipotent to 10-fold as active as all-*trans*-RA in inducing cell differentiation (Bollag and Holdener, 1992; Matsushima et al, 1992). Our data, on the other hand, show that 9-*cis*-RA is less active than all-*trans*-RA. In F9 embryonal carcinoma cells, retinol was approximately 10% as active as all-*trans*-RA in inducing differentiation (Willams and Napoli, 1985). In the clonal rhabdomyosarcoma cell line, BA-HAN-1C, the metabolites 4-keto-all-*trans*-RA and 4-hydroxy-all-*trans*-RA exhibited differentiation-inducing activity that was significantly weaker than the inducing activity of all-*trans*-RA (Ramp et al, 1994). In RbTE cells and HL-60 leukaemia cells, the biological activity of the synthetic retinoid CH55 was much higher (20–25 times) than that of all-*trans*-RA (Jetten et al, 1987). Overall, the RA catabolism-inducing capacity of the different retinoids correlates with the retinoic acid receptor binding affinities of the retinoids (Crettaz et al, 1990; Sani et al, 1990; Repa et al, 1993; Berggren Soderhund et al, 1995), suggesting that induction of RA catabolism is a receptor-mediated process. Two other lines of evidence substantiate this idea. MCF-7 cells treated with $10^{-6}$ M and $10^{-7}$ M all-*trans*-RA for 24 h showed slightly decreased CRABP-II mRNA levels in comparison to untreated cells. Induction of RA catabolism, therefore, cannot be the result of more all-*trans*-RA bound to CRABP and the ‘catalyst’ function ascribed to this binding. Secondly, induction of catabolism was inhibited by actinomycin D, indicating a transcriptional event. Therefore, preincubation with retinoids does not stabilize or activate the metabolizing enzyme molecules already present. Rather, transcription of the gene(s) for this enzyme(s) was required. The final proof for the involvement of retinoic acid receptor-mediated induction would of course be demonstration of a RARE (retinoic acid responsive element) sequence in the promotor region of the gene(s) coding for the cytochrome P450 involved in RA catabolism. Until now, however, the gene(s) has not been cloned and the exact P450 isozyme(s) responsible for this important metabolic pathway is unknown.

Pharmacological characterization of inhibition of RA catabolism was also performed. The different RA isomers all inhibited this reaction with decreasing activity in the order: all-*trans*-RA > 13-*cis*-RA > 9-*cis*-RA. Oxidation of the retinoids in the 4-position led to a decrease in activity that was more pronounced for 13-*cis*-RA than for all-*trans*-RA. Retinol was only a weak inhibitor of RA catabolism. This is somewhat surprising as one of the major pathways for oxidative catabolism of retinol is via 4-hydroxylation (Leo and Lieber, 1985). This could mean that RA is a better substrate for this enzyme than retinol or, alternatively, that each retinoid is 4-hydroxylated by different P450 (iso)enzymes. The biologically very active, synthetic retinoid CH55 did not inhibit RA catabolism at all. The activity of liarozole-fumarate (0.44 μM) was only slightly lower than that of all-*trans*-RA. This $IC_{50}$ value is similar to those reported previously for liarozole-fumarate in rat Dunning R3327G tumour homogenates (0.26 μM) and rat liver homogenates (0.14 μM) (Krekels et al, 1995). Taken together, the results presented here show that all-*trans*-RA catabolism can be induced by natural as well as synthetic retinoids and that induction is a receptor-mediated process. The induced metabolism can be inhibited by several retinoids, including the 13-*cis* and 9-*cis* isomers of RA. The data further illustrate an important difficulty in using RA for in vitro work. Unresponsiveness of cells towards RA may be the consequence of induced RA catabolism, especially during long-term experiments. Finally, this study also confirms liarozole-fumarate as an active inhibitor of all-*trans*-RA catabolism not only in the liver but also in tumour cells. This inhibition is thought to underlie the antitumoral properties of liarozole-fumarate, which is currently under clinical investigation.

### ABBREVIATIONS

RA, retinoic acid; CRABP, cytosolic retinoic acid-binding protein; $K_m$, Michaelis–Menten constant; $V_{max}$, maximal velocity of an enzyme-catalysed reaction; RAR and RXR, retinoid receptors; PSA, prostate-specific antigen; APL, acute promyelocytic leukaemia; dCTP, deoxycytidine triphosphate; GAPDH, glyceraldehyde phosphate dehydrogenase; RARE, retinoic acid responsive element; SSC, standard sodium citrate.

### ACKNOWLEDGEMENTS

We sincerely thank Dr R De Coster and Dr C Bowden for their helpful discussions and critical review of the manuscript. The technical assistance of Rita Leys and Lambert Leijssen and colleagues in the preparation of this manuscript is greatly appreciated.

### REFERENCES

Allenby G, Bocquel M-T, Saunders M, Kazmer S, Speck J, Rosenberg M, Lovey A, Kastner P, Grippo JF, Chambon P and Levin AA (1993) Retinoic acid receptors and retinoid x receptors: interactions with endogenous retinoic acids. *Proc Natl Acad Sci USA* **90**: 30–34

Berggren Soderhund M, Johanesson G and Fex G (1995) Expression of human all-trans-retinoic acid receptor beta and its ligand-binding domain in escherichia coli. *Biochem J* **308**: 353–359

Bhat PV and Lacroix A (1989) Metabolism of retinol and retinoic acid in N-methyl-N-nitrosourea-induced mammary carcinoma in rats. *Cancer Res* **49**: 139–144

Bollag W and Holdener EE (1992) Retonoids in cancer prevention and therapy. *Ann Oncol* **3**: 513–526

Brand N, Petkovich M, Krust A, Chambon P, De The M, Marchio A, Tiollais P and Dejean A (1988) Identification of a second human retinoic acid receptor. *Nature* **332**: 850–853

Crettaz M, Baron A, Siegenthaler G and Hunziker W (1990) Ligand specifities of recombinant retinoic acid receptors RAR-alpha and RAR-beta. *Biochem J* **272**: 391–397

De Coster R, Wouters W, Van Ginckel R, End D, Krekels M, Coene M-C and Bowden C (1992) Experimental studies with liarozole (R 75 251): an antitumoral agent which inhibits retinoic acid breakdown. *J Steroid Biochem Molec Biol* **43**: 197–210

Dijkman GA, Van Moorselaar RJA, Van Ginckel R, Van Stratum P, Wouters L, Debruyne FMJ, Schalken JA and De Coster R (1994) Antitumoral effects of liarozole in androgen-dependent and independent R3327-Dunning prostate adenocarcinomas. *J Urol* **151**: 217–222

Duell E, Astrom A, Griffiths CEM, Chambon P and Voorhees JJ (1992) Human skin levels of retinoic acid and cytochrome P450-derived 4-hydroxy retinoic acid after topical application of retinoic acid *in vivo* compared to concentrations required to stimulate retinoic acid-receptor-mediated transcription *in vitro*. *J Clin Invest* **90**: 1269–1274

Elder JT, Cromie MA, Griffiths CEM, Chambon P and Voorhees JJ (1993) Stimulus-selective induction of CRABP II mRNA: a marker for retinoic acid action in human skin. *J Invest Dermatol* **100**: 356–359

Fiorella PD and Napoli JL (1991) Expression of cellular retinoic acid binding protein (CRABP) in escherichia coli. Characterization and evidence that holo-CRABP is a substrate in retinoic acid metabolism. *J Biol Chem* **266**: 16572–16579

Frolik CA, Roberts AB, Tavela TE, Roller PP, Newton DL and Sporn MB (1979) Isolation and identification of 4-hydroxy and 4-oxoretinoic acid. *In vitro* metabolites of all-trans-retinoic acid in hamster trachea and liver. *Biochemistry* **18**: 2092–2097

Frolik CA, Roller PP, Roberts AB and Sporn MB (1980) *In vitro* and *in vivo* metabolism of all-trans- and 13-cis-retinoic acid in hamsters. *J Biol Chem* **255**: 8057–8062

© Cancer Research Campaign 1997

Garrabrant TA and End DW (1995) A rapid assay for measuring the metabolism of [$^{3}$H]-retinoic acid in cell cultures. *Pharmacol Toxicol Methods* **34**: 219–223

Giguere V, Ong ES, Segui P and Evans RM (1987) Identification of a receptor for the morphogen retinoic acid. *Nature* **330**: 624–629

Han IS and Choi J-H (1996) Highly specific cytochrome P450-like enzymes for all-*trans*-retinoic acid in T47D human breast cancer cells. *J Clin Endocrinol Metab* **81**: 2069–2075

Hanni R and Bigler F (1977) Isolation and identification of three major metabolies of retinoic acid from rat feces. *Helv Chim Acta* **60**: 881–887

Hanni R, Bigler F, Meister W and Englert G (1976) Isolation and identification of three urinary metabolites of retinoic acid in the rat. *Helv Chim Acta* **59**: 2221–2227

Heyman RA, Mangelsdorf DJ, Dyck JA, Stein RB, Eichelle G, Evans RM and Thaller C (1992) 9-cis-retinoic acid is a high affinity ligand for the retinoid x receptor. *Cell* **68**: 397–406

Ho CK (1985) Synergistic anticellular effect of a combination of β-interferon and retinoic acid against U937 cells. *Cancer Res* **45**: 5348–5351

Jetten AM, Anderson K, Deas MA, Kagechika H, Lotan R, Rearick JI and Shudo K (1987) New benzoic acid derivatives with with retinoid activity: lack of direct correlation between biological activity and binding to cellular retinoic acid binding protein. *Cancer Res* **47**: 3523–3527

Jones-Villeneuve EMV, McBurney MW, Rogers KA and Kalnins VI (1982) Retinoic acid induces embryonal carcinoma cells to differentiate into neurons and glial cells. *J Cell Biol* **94**: 253–262

Kraft JC, Slikker W, Bailey JR, Roberts LG, Fisher B, Wittfout W and Nau H (1991) Plasma pharmacokinetics and metabolism of β-cis and all-trans-retinoic acid in the cynomolgus monkey and the identification of β-cis and all-trans-retinoic-β-glucuronides. *Drug Metab Dispos* **19**: 317–324

Krekels MDWG, Zimmerman J, Janssens B, Van Ginckel R, Van Hove C, Coene M-C and Wouters W (1995) Analysis of the oxidative catabolism of retinoic acid in rat Dunning R3327G prostate tumors. *Prostate* **29**: 35–41

Krust A, Kastner P, Petkovich M, Zelent A and Chambon P (1989) A third human retinoic acid receptor: μ RAR gamma. *Proc Natl Acad Sci USA* **86**: 5310–5314

Lampron C, Rochette-Egly C, Gorry P, Dolle P, Mark M, Lufkin T, Lemeur M and Chambon P (1995) Mice deficient in cellular retinoic acid binding protein II (CRABP II) or in both CRABP I and CRABP II are essentially normal. *Development* **121**: 539–548

Leid M, Kastner P and Chambon P (1992) Multiplicity generates diversity in the retinoic acid signalling pathways. *Trends Biochem Sci* **17**: 427–433

Leo MA and Lieber CS (1985) New pathway for retinol metabolism in liver microsomes. *J Biol Chem* **260**: 5228–5231

Levin AA, Sturzenbecker LJ, Kazmer S, Bosakowski T, Huselton C, Allenby G, Speck J, Kratzeneisen C, Rosenberg M, Lovey A and Grippo JF (1992) 9-cis retinoic acid stereoisomer binds and activates the nuclear receptor RxR alpha. *Nature* **355**: 359–361

Lotan R and Lotan D (1981) Stimulation of melanogenesis in a human melanoma cell line by retinoids. *Cancer Res* **40**: 3345–3350

Mahler G, Verhelst J and Denis L (1993) Ketoconazole and liarozole in the treatment of advanced prostatic cancer. *Cancer Suppl* **71**: 1068–1073

Mangelsdorf DJ, Ong ES, Dyck JA and Evans RM (1990) Nuclear receptor that identifies a novel retinoic acid response pathway. *Nature* **345**: 224–229

Mangelsdorf DJ, Borgmeyer U, Heymans RA, Zhou JY, Ong ES, Oro AE, Kahizuka A and Evans RM (1992) Characterization of three RxR genes that mediate the action of 9-cis-retinoic acid. *Genes Dev* **6**: 329–344

Matsushima Y, Kawachi E, Tanaka H, Kagechika H, Hashimoto N and Shudo K (1992) Differentiation inducing activity of retinoic acid isomers and their oxidized analogues on human promyelocytic leukemia HL-60 cells. *Biochem Biophys Res Commun* **189**: 1136–1142

McCormick AM and Napoli JL (1982) Identification of 5,6-expoxy retinoic acid as an endogenous retinol metabolite. *J Biol Chem* **257**: 1730–1735

Miller VA, Rigas JR, Muindi JRF, Tong WP, Venkatraman E, Kris MG and Warrell P (1994) Modulation of all-trans-retinoic acid pharmacokinetics by liarozole. *Cancer Chemother Pharmacol* **34**: 522–526

Muindi J, Frankel JR, Miller WH, Jakubowski A, Scheinberg DA, Young CW, Dmitrowsky E and Warrell RP (1992) Continuous treatment with all-trans-retinoic acid causes a progressive reduction in plasma drug concentrations: implications for relapse and retinoid 'resistance' in patients with acute promyelocytic leukemia. *Blood* **79**: 299–303

Napoli JL (1986) Retinol metabolism in LCC-PK1 cells. *J Biol Chem* **261**: 13592–13597

Napoli JL (1993) Biosynthesis and metabolism of retinoic acid: roles of CRBP and CRABP in retinoic acid homeostasis. *J Nutr* **123**: 362–366

Ong DE and Chytil F (1983) Vitamin A and cancer. *Vitamin Horm* **40**: 105–144

Parkinson DR, Smith MA, Cheson BD, Stevenson MC and Friedmans MA (1992) Trans-retinoic acid and differentiation agents. *Semin Oncol* **19**: 734–741

Petkovich M, Brand NJ, Krust A and Chambon P (1987) A human retinoic acid receptor which belongs to the family of nuclear receptors. *Nature* **330**: 444–450

Ramp U, Gerharz CD, Eifler E, Busalski HK and Gabbert HE (1994) Effects of retinoic acid metabolites on proliferation and differentiation on the clonal rhabdomyosarcoma cell line BA-HAN-IC. *Biol Cell* **81**: 31–37

Repa JJ, Hanson KK and Clagett-Dame M (1993) All-trans-retinol is a ligand for the retinoic acid receptors. *Proc Natl Acad Sci USA* **90**: 7239–7297

Roberts AB, Lamb LC and Sporn MB (1980) Metabolism of all-trans-retinoic acid in hamster liver microsomes. Oxidation of 4-hydroxy to 4-keto-retinoic acid. *Arch Biochem Biophys* **199**: 374–383

Sani BP, Singh RK, Reddy LG and Gaub M-P (1990) Isolation, partial purification and characterization of nuclear retinoic acid receptor from chick skin. *Arch Biochem Biophys* **283**: 107–113

Sietsema WK and De Luca HF (1982) Retinoic acid 5,6-epoxidase. *J Biol Chem* **257**: 4265–4270

Smets G, Van Ginckel R, Daneels G, Moeremans M, Van Wauwe J, Coene M-C, Ramaekers FCS, Schalken JA, Borgers M and De Coster R (1995) Liarozole, an antitumor drug, modulates cytokeratin expression in the Dunning AT-6sq prostatic carcinoma through *in situ* accumulation of all-trans-retinoic acid. *The Prostate* **27**: 129–140.

Sporn M and Roberts AB (1983) Role of retinoids in differentiation and carcinogenesis. *Cancer Res* **43**: 3034–3040

Strickland S and Mahdavi V (1978) The induction of differentiation in teratocarcinoma stem cells by retinoic acid. *Cell* **15**: 393–403

Vanden Bossche H and Willemsens G (1990) Retinoic acid and cytochrome P450. In *Retinoids: 10 years on*, Saurat JH (ed.), pp. 79–88. Karger: Basle

Van Wauwe JP, Coene M-C, Goossens J, Van Nyen G, Cools W and Lauwers W (1988) Ketoconazole inhibits the *in vitro* and *in vivo* metabolism of all-trans-retinoic acid. *J Pharmacol Exp Ther* **245**: 718–722

Van Wauwe JP, Coene M-C, Goossens J and Monbaliu J (1990) Effects of cytochrome P450 inhibitors on the *in vivo* metabolism of all-trans-retinoic acid in rats. *J Pharmacol Exp Ther* **252**: 365–369

Van Wauwe JP, Van Nyen G, Coene M-C, Stoppie P, Cools W, Goossens G, Borghgraef P and Janssen PAJ (1992) Liarozole, an inhibitor of retinoic acid metabolism, exerts retinoid-mimetic effects *in vivo. J Pharmacol Exp Ther* **261**: 773–779

Van Wauwe JP, Coene M-C, Cools W, Goossens J, Lauwers W, Le Jeune L, Van Hove C and Van Nyen G (1994) Liarozole-fumarate inhibits the metabolism of 4-keto-all-trans-retinoic acid. *Biochem Pharmacol* **47**: 737–741

Varani J, Gendimenico GA, Shah B, Gibbs D, Capetola RJ, Mezick JA and Voorhees JJ (1991) A direct comparison of pharmacologic effects of retinoids on skin cells *in vitro* and *in vivo. Skin Pharmacol* **4**: 254–261

Williams JB and Napoli JL (1985) Metabolism of retinoic acid and retinol during differentiation of F9 embryonal carcinoma cells. *Proc Natl Acad Sci USA* **82**: 4658–4662

Williams JB and Napoli JL (1987) Inhibition of retinoic acid metabolism by imidazole antimyotics in F9 embryonal carcinoma cells. *Biochem Pharmacol* **36**: 1386–1388

Williams JB, Smilds CO, Brettel LM and Napoli JL (1987) Assessment of retinoid-induced differentiation of F9 embryonal carcinoma cells with an enzyme-linked immunosorbent assay for laminin; statistical comparison of dose-response curves. *Anal Biochem* **160**: 267–274

Wolf G (1984) Multiple functions of vitamin A. *Physiol Rev* **64**: 873–937

Wouters W, Van Dun J, Dillen A, Coene M-C, Cools W and De Coster R (1992) Effects of liarozole, a new antitumoral compound, on retinoic acid induced inhibition of cell growth and on retinoic acid metabolism in MCF-7 breast cancer cells. *Cancer Res* **52**: 2841–2846

© Cancer Research Campaign 1997